# Operation Rate for Displaced Distal Radius Fractures in the Elderly Decreased by 68% After the Implementation of Evidence-Based Practice

**DOI:** 10.7759/cureus.81452

**Published:** 2025-03-30

**Authors:** Emil Ø Nielsen, Stig Brorson, Jeannette Ø Penny, Tommy H Jensen, Thomas J Sørensen, Dennis W Hallager

**Affiliations:** 1 Department of Orthopedic Surgery, Zealand University Hospital, Køge, DNK

**Keywords:** behavior change, clinical guidelines, distal radius fractures, evidence-based clinical practice, implementation research, surgical and non-surgical treatment, trauma and orthopedic surgery

## Abstract

Background

The increasing demand for orthopedic trauma surgery resources highlights the need for an efficient approach to implementing scientifically based interventions and de-implementation of interventions already in use that have been found no better than non-surgical treatments. Several factors have been identified as barriers or facilitators for translating evidence into clinical practice and behavioral changes. To facilitate a structured approach to applying these factors, we have adapted the generic theoretical domains framework (TDF) into the “CEBO model,” providing a practical framework for implementing evidence in clinical decision-making in the hospital department of Zealand University Hospital, Koege, Denmark.

Aim

This study aims to evaluate the feasibility of the CEBO model for facilitating surgeon behavior change in an orthopedic surgery department. We will present clinical decision-making regarding the treatment of dorsally displaced distal radius fractures (DDDRF) in the elderly as an example.

Methods

Our department's standard of care for DDDRF in the elderly was surgical treatment. Under the evolving body of evidence, the CEBO model was applied to facilitate change in treating surgeons’ behavior toward the increased use of non-surgical care. Following the four steps of the CEBO model, 1) leadership support was obtained, and relevant evidence was disseminated to all colleagues across the department; 2) stakeholders were invited to a symposium discussing best evidence and future practice; 3) conclusions from the symposium were summarized in a local clinical guideline stating non-surgical treatment as a new department standard and 4) to monitor the changes in treatment, patient charts were retrospectively reviewed from one year before and after the application of the CEBO model.

Results

In the first period, 95 of 120 (79%) were surgically treated, compared to 16 of 146 (11%) in the second period. An absolute decrease in the operation rate of 68% was observed.

Conclusion

We found the CEBO model highly feasible for facilitating surgeon behavior change in our orthopedic surgery department. Future studies will investigate the model's feasibility in other institutions and compare it to other behavior-targeted interventions.

## Introduction

An evidence-practice gap can emerge if clinical practice deviates from the best evidence. This may include the need for more adaptation to new treatments or technologies or reluctance to change existing practices based on new evidence [1]. Orthopedic surgeons have previously shown reservations about changing their current practice even if faced with high-quality evidence [2,3]. Thus, more than awareness of the evidence is often required. Several other factors have been identified to influence clinician behavior as barriers to changing clinical practice, such as lack of support from peers or superiors and inadequate resources [4-6]. 

At the Centre for Evidence-Based Orthopedics (CEBO), we have developed the “CEBO model” to facilitate clinician behavior change in a clinical department, which considers these factors through a structured framework [7,8]. 

The model utilizes the theoretical domains framework (TDF) to identify barriers and facilitators [9], which can be categorized as components of capability, opportunity, or motivation concerning behavior (the COM-B system) [10]. It consists of four phases: 1) planning, 2) a symposium for stakeholders, 3) implementation and behavior design efforts, and 4) evaluation [7]. 

Distal radius fractures are common in patients aged 60 years and above [11]. Over 22 years, the surgical treatment rate has increased threefold in Denmark for young and older adults aged 60 years and above [11]. This increase seems unaffected by the increasing body of evidence, including systematic reviews of randomized controlled trials (RCTs), which have emerged since 2015. These reviews report no clinically meaningful benefit of surgical treatment in the elderly population [12-14].

This study aims to assess the feasibility of the CEBO model in influencing surgeon behavior by evaluating changes in the surgical treatment rate of DDDRF in elderly patients. The study will measure the extent of practice change, sustained adherence, and potential for broader implementation in orthopedic surgery.

## Materials and methods

Manuscript preparation was guided by the Standards for Quality Improvement Reporting Excellence (SQUIRE) guidelines for reporting quality improvement studies in healthcare [15]. 

Phases of the CEBO model

Phase 1

To compare our current practice with what is indicated by the best evidence, we used available national and international guidelines that derive from countries we usually compare with regarding standard treatments [13,16-18]. The Cochrane Library and PubMed were searched for systematic reviews [14,19]. Our clinical question was defined as whether surgical treatment of DDDRF in patients aged 60 years and older provides a clinically meaningful benefit compared to non-surgical treatment at one year in terms of functional outcome assessed using patient-reported outcome measures (PROMs). 

Our baseline practice was determined by a chart review in an electronic health record system covering eastern Denmark. In Denmark, injuries are treated by the public healthcare system available for all citizens. All patients aged 60 years or older referred for treatment of DDDRF at the hospital department of Zealand University Hospital, Koege, Denmark, from February 1, 2019, to January 31, 2020, were recorded [20]. DDDRF was characterized by the presence of at least one of the following radiological criteria [18]: 1) angulation of the distal radial articular surface exceeding 10 degrees dorsally in the sagittal plane relative to a line perpendicular to the radial longitudinal axis; 2) ulnar variance exceeding 2 mm; 3) articular step-off exceeding 2 mm; and 4) distal radioulnar joint incongruity.

Our department head supported us and invited all department surgeons to an in-house symposium to discuss future practices.

Phase 2

The collected evidence base was emailed to all surgeons, who were encouraged to suggest additional relevant references. We conducted a two-hour symposium with brief presentations of the sources of evidence and subsequent discussion of the results and their potential impact on our future practice. All participants were encouraged to ask questions and raise their concerns. This was done orally and not anonymously. A moderator kept the discussion within the confines of the evidence base that all had had the opportunity to appraise. A summary of the conclusions was documented.

Phase 3

A committee consisting of senior and junior colleagues under the supervision of the department professor formulated a guideline based on input from the symposium debate. It was encouraged that the guideline included shared decision-making. The guideline was subsequently disseminated to all department surgeons, published in the local guideline repository, and repeatedly disseminated at morning conferences. Behavioral design was planned to increase adherence to the guideline. This included readily available standard phrases in our electronic health records for surgeons to use at the initial patient assessment and first outpatient clinic visit.

Phase 4

To evaluate practice change, we performed another chart review of patients aged 60 years or older referred for DDDRF treatment from February 1, 2022, to January 31, 2023. 

Both chart reviews included all patients aged 60 or older diagnosed with the International Classification of Diseases (ICD-10; DS524, DS525, DS525A-C, DS526, DS527, DS528, DS529). The first author, an orthopaedic resident, re-evaluated all radiographs and performed chart reviews. Patients with persistent dorsally displaced distal radius fractures following closed reduction attempts in the emergency department were included. If patients had bilateral displaced DRF, they were considered two individual fractures. Treatment was considered non-operative if the patients had not received surgical treatment at any public institution in eastern Denmark or had been referred for surgical treatment at a private clinic within four weeks of diagnosis.

Statistics

As the study only concerns the involved surgeons' behavior in one department, we do not claim inference. Thus, data is presented using descriptive statistics only.

Ethics, registration, data sharing plan, funding, and potential conflicts of interest

The study was approved by our institutional review board on 7-11-2022. As a quality improvement study on surgeon behavior using retrospective patient data, it is exempt from other institutional approval or informed consent. As per local policy, patient charts were checked for explicit statements about declined acceptance for participation in quality assessments. The study has not been pre-registered. Data collection consists of patient age, sex, and treatment status. These data are available without patient identifiers by request to the corresponding author up to 10 years from study approval. The authors declare that they have no conflicts of interest concerning this work. No funding was received for this work.

## Results

Local guideline

The new local guideline stated that evidence failed to show clinically meaningful benefits of surgery compared to non-surgical treatment in terms of function, quality of life, and pain after one year. Thus, the new first-line treatment was the non-surgical treatment of DDDRF. The guideline did not apply to patients with concomitant or open fractures (Gustilo grade >1) [21], high-energy trauma, neurovascular compromise, or patients relying on a walking aid in the injured hand. Moreover, patients with persistent or worsening pain at follow-up should be considered for surgery. Finally, after being informed of the expected functional outcomes from each treatment option, the patient was to make the final treatment decision.

Practice change

Following the application of the CEBO model, we observed a 68% absolute reduction in the surgical management of DDDRF in the elderly over a one-year period, which corresponds to a change from surgical treatment of 79% of 120 patients with a mean age of 71 years before utilization of the CEBO model to 11% of 146 elderly patients with a mean age of 75 years during the second period (Table 1). According to patient charts, the main reasons for non-surgical management before the implementation were patients rejecting surgery, patients not fit for surgery, or low functional demand. 

**Table 1:** Characteristics of the included fractures in each period. DRF: distal radius fractures

Time	2019-2020	2022-2023
Dorsally displaced DRF in patients older than 60 years	120	146
Surgical treatment	95 (79%)	16 (11%)
Proportion of women	88%	95%
Mean age of patients in years and range	71 (60-96)	76 (60-93)

At a follow-up after the implementation, reasons for surgery were patient preference, severe pain on the first follow-up visit in the outpatient clinic, patients having bilateral fractures, patients wanting faster recovery, and surgeons recommending surgery based on fracture configuration on radiographs.

## Discussion

Following applying the CEBO model in our department, we observed a decrease in the surgery rate from 79% to 11% in patients aged 60 and older. 

This study suggests that the CEBO model may be a feasible tool for behavior change in orthopedic surgeons. The findings align with our previous experience with the model [8]. To overcome possible changes in patient demographics, fracture incidence, and surgical treatment incidence, the two inclusion periods were chosen to precede and succeed the community lockdowns due to the COVID-19 pandemic. Thus, the one-year follow-up period was postponed starting 15 months after the symposium. Postponing the follow-up showed that behavior change was sustained for at least two years, indicating an integration of the practice change in the organization. Moreover, no trend in a quarter-by-quarter analysis of surgical treatment rate was seen. This is despite an increased incidence of referred patients with DDDRF compared to the period before the pandemic. 

We considered the lack of awareness of the conclusions in the literature and the potential lack of peer support as a significant barrier to practice change. In our institution, junior colleagues primarily manage patients presenting with acute fractures under the supervision of more experienced surgeons. The fellowship training requires frequent rotations between orthopedic departments; thus, junior surgeons could face conflicting practices. We found the model able to overcome these barriers. We consider the open discussion a critical element of the model, as it allows inferring ownership to stakeholders regarding future practice and the new local guidelines. We also suggest that the discussion influence surgeons’ beliefs and motivate their adherence to the change of practice.

Treatment choice before this intervention was mainly based on radiological criteria. Patient preferences and values were sparsely investigated. Patients should be more involved in decision-making. The guideline is based on the evidence pillar of evidence-based practice [22]. The remaining two pillars, clinical experience and patient preferences and values, remain context and patient-specific but of equal importance.

We acknowledge that the preferred treatment of DDDRF in the elderly population is under debate. This strengthens our belief in the CEBO model's ability to facilitate behavior change at a department level despite external controversy.

Limitations

The development of the CEBO model in our department could predispose its acceptance and integration at our institution. The observational design does not allow comparison with other behavior change interventions; however, the current study aimed to assess the feasibility before considering trials involving other institutions and comparisons. The desired behavior in this example was less resource-consuming. It did not require new skills or organizational changes, which are all factors that could influence the ability to get leadership support and adherence to the target behavior.

A further limitation of this study is that all radiographs and chart reviews were evaluated unblinded by the first author only, and inter-rater reliability was not assessed. Although shared decision-making was encouraged in the guideline, this study did not evaluate how the local guideline has been applied in every patient encounter or how patients perceive it. 

We observed a five-year difference in mean patient age from the first to the second cohort. As functional demands generally decrease with age, this may have influenced the surgery rates and thus may be a confounding factor. Other unmeasured confounders may have had a similar impact, and these should be accounted for in future studies examining the efficacy of the CEBO model.

## Conclusions

We found the CEBO model a feasible tool for implementing evidence-based practice among surgeons in an orthopedic department. We observed a pronounced change in practice that persisted over at least two years. Further investigations of the feasibility and efficiency of the model in other institutions and comparison to other behavior-targeted interventions remain to be evaluated in future studies.
